# A Study of the Reinforcement Effect of MWCNTs onto Polyimide Flat Sheet Membranes

**DOI:** 10.3390/polym12061381

**Published:** 2020-06-19

**Authors:** Andreas A. Sapalidis, Panagiotis I. Karantzis, Achilles Vairis, Stephanos F. Nitodas, Stéphan Barbe, Evangelos P. Favvas

**Affiliations:** 1 Membranes and Materials for Environmental Separations Laboratory, Institute of Nanoscience and Nanotechnology, NCSR “Demokritos”, Ag. Paraskevi, 15341 Attica, Greece; pan.karantzis@gmail.com; 2 Department of Mechanical Engineering, Hellenic Mediterranean University, 71410 Heraklion Crete, Greece; vairis@hmu.gr; 3 Department of Materials Science and Chemical Engineering, Stony Brook University, Stony Brook, NY 11794, USA; steve.nitodas@stonybrook.edu; 4 Technische Hochschule Köln, Faculty of Applied Natural Sciences, Kaiser-Wilhelm-Allee, Gebäude E39, 51373 Leverkusen, Germany; stephan.barbe@th-koeln.de

**Keywords:** polymer nanocomposite materials, mixed matrix membranes, carbon nanotubes, mechanical properties, thermal properties, gas permeability

## Abstract

Polyimides rank among the most heat-resistant polymers and find application in a variety of fields, including transportation, electronics, and membrane technology. The aim of this work is to study the structural, thermal, mechanical, and gas permeation properties of polyimide based nanocomposite membranes in flat sheet configuration. For this purpose, numerous advanced techniques such as atomic force microscopy (AFM), SEM, TEM, TGA, FT-IR, tensile strength, elongation test, and gas permeability measurements were carried out. In particular, BTDA–TDI/MDI (Ρ84) co-polyimide was used as the matrix of the studied membranes, whereas multi-wall carbon nanotubes were employed as filler material at concentrations of up to 5 wt.% All studied films were prepared by the dry-cast process resulting in non-porous films of about 30–50 μm of thickness. An optimum filler concentration of 2 wt.% was estimated. At this concentration, both thermal and mechanical properties of the prepared membranes were improved, and the highest gas permeability values were also obtained. Finally, gas permeability experiments were carried out at 25, 50, and 100 °C with seven different pure gases. The results revealed that the uniform carbon nanotubes dispersion lead to enhanced gas permeation properties.

## 1. Introduction

Membrane technology provides attractive advantages over the “traditional” absorption, and adsorption, processes which suffer from drawbacks like corrosivity, complex process lay-out, high installation and operation costs, and energy-consuming regeneration processes [1]. Nowadays, membrane technology has received ample attention in various industries towards effective gas separation processes that mostly rely on low energy consumption, high efficiency, stability, and easy processability [2], as well as potential use for dual conversion and capture [3]. Organic polymeric membranes and inorganic polycrystalline membranes are commonly used for gas separation processes. In this regard, emphasis has been given on CO_2_ separation process and both systems present many advantages but also limitations. Inorganic membranes possess high thermal, mechanical, and chemical stability combined with long-lasting durability. Compared to polymeric membranes, they exhibit higher permeability and selectivity due to their textural features [4]. On the other hand, parameters such as the extremely, sometimes, high cost, brittleness, difficulties in membrane processability, and scale-up potential rein in the commercialization of inorganic membranes [5,6].

In the past years, considerable effort has been dedicated to the development and enhancement of hybrid membrane materials [7,8] by the incorporation of nanoparticles [9]. The interest in such polymer modification is connected with the improvement of the membrane properties, such as permeability coefficients, selectivity, and mechanical properties [10]. These effects are correlated to the change of the polymer chain mobility, the degree of crystallinity, and the structure of polymer free volume, which interacts with nanoparticles [11]. The change of permeation is connected with the formation of a percolation cluster in the nanostructured domains at a critical concentration of nanoparticles, such as multi-wall carbon nanotubes (MWCNTs), in the polymer [12].

Polymer nanocomposite materials have drawn extensive attention due to their capability of tuning their physicochemical properties depending on the application. During the development of polymer nanocomposites, the choice of both polymer type and appropriate nanofiller, combined with the employed processing parameters, can result in new materials with completely different properties that can be used in a wide range of different applications. In recent years, polymeric nanocomposites have had a broad use in: (1) the biomedical field [13], (2) the textile industry [14], (3) the field of packaging materials [15], (4) the field of electronics [16], and (5) the field of engineering constructions, like automotive and building materials and aerospace parts. [17]. Among these fields the membrane technology has embraced the concept of nanocomposite materials by introducing the definition of mixed matrix membranes (MMMs). 

Mixed matrix membranes are the “plan B” of the traditional polymeric membranes, as they could redress the drawback of the Robeson “trade-off” effect in commercial membranes [18]. There are several known factors that influence the performance of the MMMs during the gas separation processes. The most significant ones are: (1) the nature of the polymer matrix, (2) the structural and the physicochemical properties of the fillers, (3) the formatted filler-matrix interaction, and (4) the membrane preparation-processing methods. Among these factors, the two crucial parameters for developing efficient MMMs are the selection of the filler material and the polymer matrix. With reference to a particular membrane polymer material, the selection of an appropriate filler material could be of the highest importance because fillers might exhibit a weak adhesion to the polymer matrix, and, finally, lead to non-uniform dispersion and marginal improvements or even worst membrane mechanical structure and poor performance in gas separation processes [19]. Therefore, the chosen filler material should be compatible with the polymer matrix. The existing chemistry and the roughness of the filler material’s surface play a determinant role in the final formed structure and properties of the nanocomposite material [20]. Porous materials satisfy these two prerequisites and can be used as promising membrane filler materials. Well-known types of porous filler materials are: Mesoporous silica [21], zeolites [22], carbon nanotubes (CNT) [23,24], graphene oxide (GO) [25,26], and metal-organic frameworks (MOFs) [27,28]. 

In the present study, MWCNTs were used as fillers in polyimides (BTDA-TDI/MDI (P84)) aiming at improving their mechanical and gas permeability efficiency and mechanical durability. During the last decade, carbon nanotubes have attracted considerable attention, in a commercial scale, because of their impressive mechanical and electrical properties, which have created a potential for future applications [29]. Although single wall carbon nanotubes (SWCNTs) are superior to MWCNTs in their electrical and heat conductivity, MWCNTs are usually preferred, especially in engineering applications, mainly due to their facile production, better mechanical properties, and low cost of supply. However, the MWCNTs use is still not applicable, even at relatively low loadings, because of their poor processability, mainly due to the formation of agglomerates [30]. Carbon nanotubes have a continuous network of sp^2^ hybridized carbon atoms forcing them to stack strongly via Van der Waals forces [31]. Therefore, when carbon nanotubes are mixed with liquids (solvents, resins, or even polymer melts), they tend to precipitate and/or form aggregates; as a consequence, the addition of carbon nanotubes does not result in any enhancement of the final properties of the materials. This enhancement can be achieved by chemical functionalization of the outer surface [23,32,33,34] of the MWCNTs or simpler by using high energy probe sonication into the MWCNTs/solvent system, with the best results obtained by combining these two approaches. In regard to the functionalization, the degree of modification achieved on the surface of MWCNTs plays a key role, and high degrees of modification may lead to an adverse effect on the nanotubes properties.

Polyimides (PI) are high-performance polymers of imide monomers which contain two acyl groups (C=O) bonded to nitrogen (N). These polymers are known for their high temperature performance, in the 200–300 °C range, as well as chemical resistance [35]. They are employed to replace the conventional use of glass, metals, and, even, steel in a broad range of industrial applications, including membrane materials for separation [36], high temperature plastics, adhesives, dielectrics, photoresists, sensors, high temperature fuel cells, flat panel displays, aerospace applications, and chemical/petrochemical industries [37].

Carbon nanotubes have been recently studied as fillers for polyimides because their addition in PI is expected to enhance mechanical and electrical properties while improving load transfer and tear resistance [12]. The combination of carbon nanotubes and polyimides is deemed to play an important role in the development of novel high-performance nanocomposites [10,38]. However, most of the studies to date focus on electrical/electronic applications and it was shown that CNTs may be able to achieve certain levels of electric conductivity through a percolation network for charge mitigation and electromagnetic shielding [39,40]. Results on mechanical and/or thermal properties of CNTs-PI composites are rather limited in these studies. In addition, mostly functionalized carbon nanotubes were utilized in order to avoid CNTs agglomeration during the composites synthesis [41]. In the present study, the approach of pristine MWCNTs was employed in an effort to develop protocols for the efficient dispersion of nanotubes at a lower cost.

## 2. Materials and Methods

### 2.1. Materials

**Figure 1:**
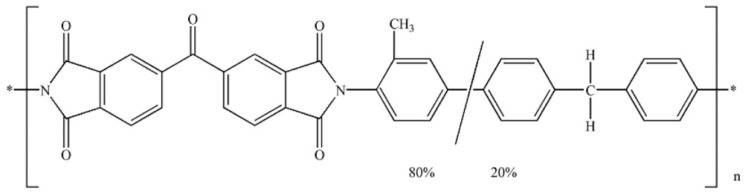
Chemical structure of BTDA–TDI/MDI (Ρ84) co-polyimide.

The commercial co-polyimide (3,3′4,4′-benzophenone tetracarboxylic dianhydride and 80% methylphenylene-diamine + 20% methylene diamine), BTDA–TDI/MDI (Ρ84) co-polyimide (Figure 1), in powder form, with a MW of 60,000 g/mol, obtained from Ciba Geigy, Basel, Switzerland, was used as the polymer for the preparation of both single phase and composite films. BTDA–TDI/MDI (Ρ84) co-polyimide is a soluble thermoplastic polyimide that is fully imidized, does not require high temperatures for processing, presents excellent adhesion, and renders durable and tough films. Finally, it is characterized by excellent chemical resistance and provides very good thermal properties [42] while it is soluble in a variety of common solvents, such as dimethylformamide (DMF) and N-methyl-2-pyrrolidone (NMP).

In the current study NMP was used as the polymer solvent.

The filler material was multi-wall carbon nanotubes with a purity of 95% [43]. The morphological properties of the carbon nanotubes were evaluated by scanning electron microscopy (JSM 7401F, Jeol, Akishima City, Japan) and transmission electron microscopy (JEM-2100). Two types of ultrasonic devices were used to treat the solutions: A Branson 3800 bath ultrasonic cleaner and a Hielscher Lab tip ultrasonic processor.

### 2.2. Fabrication of MWCNTs/Polyimide Nanocomposite Films

Two types of membranes, were prepared: Non-porous polyimide films and non-porous nanocomposite MWCNTs/polyimide films. The procedure for the polyimide film preparation encompasses the following steps: 5 g of the polymeric powder was dissolved into 100 mL NMP and the solution was mixed for about 4 h at 55 °C. Subsequently, the solution was stabilized by bath sonication for 3 h at 55 °C. Finally, 9 mL of the solution was poured into a glass petri dish (Pyrex^®^) of 12 cm diameter and left for 48 h into the oven at 75 °C. The average thickness of the produced films was 50 μm.

In the case of nanocomposite MWCNTs/polyimide films the procedure applied was the following: 1 g of MWCNTs was dispersed into 100 mL NMP using a tip sonicator for about 15 min, at 50 °C. The use of a high energy tip sonicator was necessary in order to break the carbon nanotubes bundles, which are strongly formed due to the π-π interaction, which is more competitive than that of the polymer matrix [44]. By mixing the above MWCNTs’ solution and the 5% *w/v* solution of polyimide into NMP, which was used in the single phase films preparation, a series of solutions of 5% of polyimide and 1%, 2%, 3%, 4%, and 5% of MWCNTs into NMP were produced. The mixing took place with a mechanical stirrer for 4 h at 55 °C for each sample.

Subsequently, a mild mixing in a sonicator bath was applied for 20 min and finally 10 mL of each solution of MWCNTs/polyimide/NMP was cast into a glass petri dish (Pyrex^®^) of 12 cm diameter and placed into the oven to dry for 48 h at 75 °C. The average thickness of the produced films was 40 μm. The coded produced films are presented at Table 1.

### 2.3. Characterization Techniques

Both single phase and composite films were characterized using a variety of techniques. The surface and cross-section structural characteristics of the membranes were evaluated by scanning electron microscopy (Jeol-JSM-7401F Field Emission SEM, Tokyo, Japan). Prior to imaging, the samples were cut into 0.5 by 0.5 cm pieces and placed on a metallic substrate, fixed with carbon tape, and, finally, gold-sputtered. The topography of the surface was determined by atomic force microscopy (AFM). For this purpose, a Veeco Innova, using Bruker RTESTPA-300 probes (3601 Calle Tecate Suite C, Camarillo, CA 93012, U.S.A.) in non-contact (tapping) mode, was employed. 

The thermal properties of the studied membranes were investigated in a TGA/DTA-DSC Thermogravimetric-Differential Thermal Analyzer (Setaram, Caluire-et-Cuire, France, Setsys Evolution 18). Samples of a mass of about 7 mg were heated from ambient temperature up to 900 °C in platinum crucibles, under a constant flow rate of 20 cm^3^/min pure Argon. The temperature increased from 25 °C to 120 °C in a rate of 2.5 °C/min, until 120 °C, where they remained for 30 min in order to remove any absorbed water molecules and volatile impurities. Afterwards the same heating rate was applied up to 900 °C. The samples were also tested by differential scanning calorimetry (DSC) in a TA Instruments, Model MDSC 2920 (New Castle, DE, USA). The runs were conducted using a heating ramp of 2 °C/min, and a cooling rate of 5 °C/min with a temperature modulation of ±0.32 °C every 60 s.

Fourier-transform infrared spectroscopy (FTIR) spectra of all polyimide films were recorded using a horizontal attenuated total reflectance ATR trough plate crystal cell (Thermo Electron 6700 ATR diamond) with a Nicolet 6700 FTIR (Thermo Electron Corporation, Waltham, MA, USA) operating at room temperature. For the spectra analysis, the samples were placed on the crystal cell, which was in turn, mounted onto the spectrometer. The spectra were collected in the range of 400–4000 cm^−1^. The background spectrum was recorded at room temperature with an empty cell. 

The tensile strength, elongation at break, and Young’s modulus were measured in a Thümler GmbH, Nurnberg Germany, Z3 tensile tester equipped with a 250 N Nordic transducer load cell. Rectangular shaped samples (15 mm by 120 mm) were tested with an extension rate set to 50 mm/min, according to ASTM D-882. The results represent the average of five samples, while prior to the measurement the specimens remained at ambient environment (~23 °C and ~50% rh) for 48 h.

Oxygen permeability in controlled humidity environment was determined in a PBI-Dansensor OPT-5000 permeability tester coupled with a Julabo F12-MC refrigerated-heating circulator.

Permeation measurements of various pure gases (He, H_2_, CO_2_, O_2_, CO, N_2_, and CH_4_) were performed using the variable pressure method in a low vacuum/high-pressure stainless steel permeation rig [45]. The effective permeation area for the polyimide flat sheet membrane modules was approximately 7 cm^2^. Due to the temperature limited performance of the epoxy resin (up to 120 °C) all the permeability experiments were performed at temperatures up to 100 °C. Gas was admitted to the high-pressure section of the rig, while the low-pressure side remained isolated under vacuum. Permeance experiments were performed by continuously monitoring the pressure increase in the low-pressure side of the rig by means of an accurate differential pressure transducer. The permeability, *P*, (Barrer) is defined as the gas flow rate multiplied by the membrane thickness and divided by the pressure difference across the membrane and by the area of the membrane, as following:

(1) $$Permeability\;\left(P\right)=\frac{flow\;rate\times thickness}{area\times pressure\;difference}$$

while the Barrer unit is defined as:

(2) $$P\left(1\;\mathrm{Barrer}\right)=10^{-10}\times \frac{cm^{3}\left(STP\right)\times cm}{cm^{2}\times s\times cmHg}$$

and the ideal selectivity is calculated from the single gas permeability values by using the following simplified equation:

(3) $$\alpha_{ideal\left(\frac{H_{2}}{CH_{4}}\right)}=\frac{P_{H_{2}}}{P_{CH_{4}}}$$

Furthermore, oxygen permeability measurements were performed from dry environment up to 85% relative humidity in a special designed apparatus [46,47].

## 3. Results

BTDA–TDI/MDI (Ρ84) and MWCNTs composites were prepared as described above. A pure polyimide film was also prepared in order to compare its properties with those of nanocomposites. Both pure and mixed matrix membranes, with a thickness of about ~40 μm were characterized. More specifically, DSC, TGA, SEM, AFM, and FTIR were used for the physicochemical characterization and the investigation of their properties. 

### 3.1. Morphology of MWCNTs and Nanocomposite Materials

**Figure 2:**
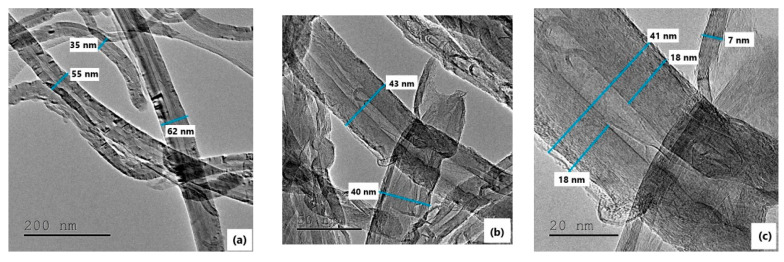
Low (**a**) and high magnification (**b** and **c**) TEM images of raw MWCNTs.

Both AFM and SEM were used for the investigation of the morphological characteristics, the surface homogeneity, the existence of cracks and defects, and the verification of the thickness of the prepared nanocomposite films. In addition (Figure 2), transmission electron microscopy (TEM) was used for the estimation of the dimensions of MWCNTs. TEM images of the MWCNTs used as fillers are depicted in Figure 2. These images clearly show that the employed material consisted of pure MWCNTs with a narrow size distribution and uniform delimitation of the building walls. Overall, fluctuation in nanotube size, external diameter, in a range from 35 to 62 nm was observed.

From Figure 2c, high magnification view, an average diameter of the MWCNT of approximately 40 nm was estimated. Another interesting finding is that the main body of the carbon nanotubes structure appears to be built from concentric carbon walls and only one open channel (pore) with a diameter of approximately 5 nm was observed at the MWCNTs’ center. The total wall thickness was about 36 nm. 

The purity of the nanotubes was high as depicted in the TEM images, where neither MWCNTs’ agglomerations and amorphous carbon nor catalyst particles were present. Information related to the roughness of the membranes’ surface can be deduced from the AFM images. PIMWNT2 and PIMWNT5 membranes were chosen indicatively in order to estimate the impact of the filler carbon nanotubes concentrations.

**Figure 3:**
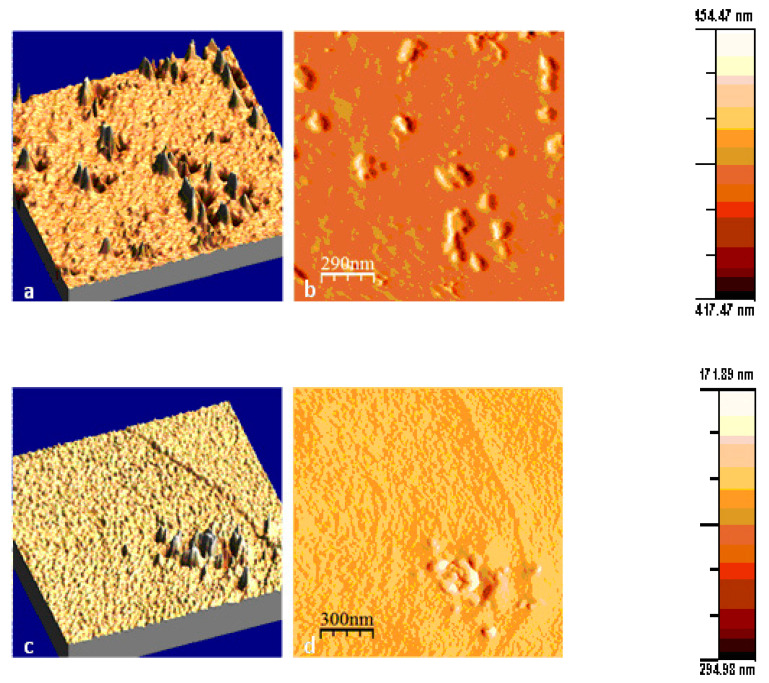
Atomic force microscopy (AFM) images of PIMWNT5 (**a,b**) and PIMWNT2 (**c,d**) membranes.

Figure 3 shows the AFM images at two of the studied films, this with lower MWCNTs concentration, 2% wt (PIMWNT2), and the PIMWNT5 film with the higher MWCNTs concentration, 5% wt (PIMWNT5). For both samples, the 2D images (Figure 3) evince a smooth overall surface with few projections from the filler MWCNT materials. From a wide mapping of the samples, no cracks and defects were observed even in the case of the film with the higher MWCNTs concentration.

**Figure 4:**
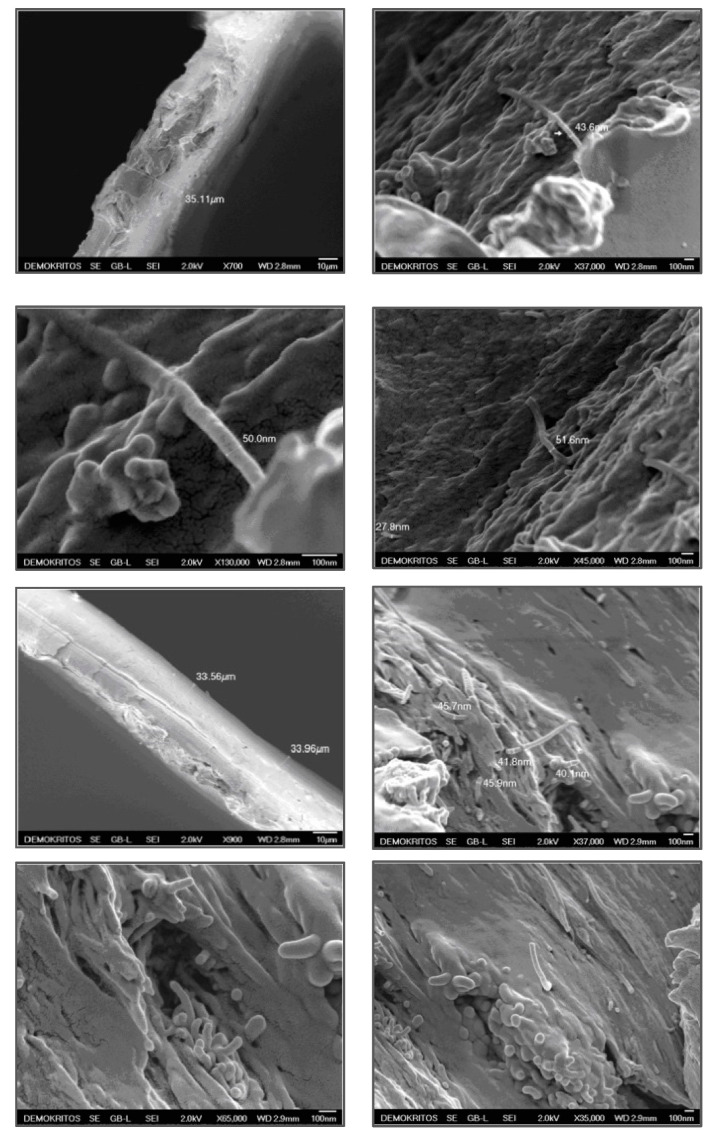
SEM images of PIMWNT2 (upper 2 rows) and PIMWNT5 (bottom 2 rows) mixed matrix membranes.

Scanning electron microscope images were obtained for the same selected membranes. The SEM images show a homogeneous surface and no cracks (Figure 4). The presence of aggregates, the number and size of which was considered not to be prohibitive, was found to be characteristic of the membranes at higher MWCNTs concentrations, as in the case of PIMWNT5. The average thickness of both membranes was about 35 μm and characterized by a dense continuous structure. The clear presence of carbon nanotubes in the cross section, without any observable agglomeration points, reveals evidence for a high dispersion grade. The dimension of the MWCNTs remains in the size range estimated by TEM and suggests that there is good adhesion of the carbon phase and the polymeric chains and that no coating of the polymer on the carbon nanotubes occurred. This was also confirmed by permeability experiments where the presence of the filler materials led to the measurement of higher gas permeability values with mixed matrix membranes.

### 3.2. Thermal Analysis

For the thermogravimetric analysis, the membranes were cut into small specimens and an amount of ~7 mg was placed in the instrument’s crucible, using a heating rate of 2.5 °C/min, in argon atmosphere, from ambient temperature till 900 °C.

**Figure 5:**
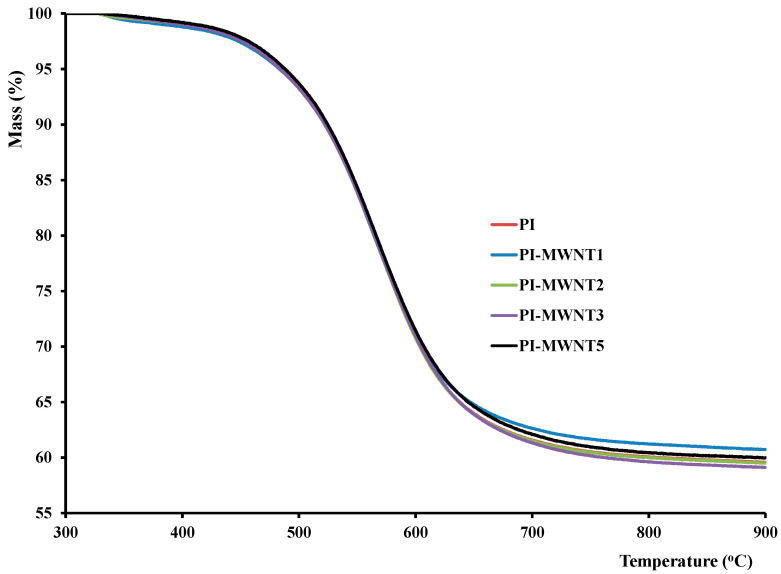
TGA curve for a pure polyimides (PI) membrane and PI-MWCNTs membranes.

From the curve of Figure 5 it is observed that the membranes show one stage of mass loss which initiates above 400 °C and is due to the degradation of the polymer, as also observed by other studies [48]. The graph of Figure 5 also shows that there is negligible effect from the presence of carbon nanotubes in the polymer matrix, on the overall thermal stability of the polymer.

The greater overall mass loss observed in MWCNTs-polymer samples than in pure polyimide can be explained by a possible higher solvent residual content in these samples. Table 2 also shows a similar trend in the loss of the membrane mass. 

According to the DSC measurements, it can be concluded that the presence of carbon nanotubes into the membrane matrices provides better thermal properties. Specifically, the *T_g_* of the PI membranes was measured 325 °C and increases slightly depending on the filler concentration. There was an increase of one degree Celsius by the addition of 1 wt.% of MWCNTs, whereas by the addition of 2 wt.% the *T_g_* was recorded 329 °C, which is the highest glass transition temperature recorded for all studied membranes. In the cases of the PIMWNT3 and PIMWNT5 membranes, the *T_g_* decreased to 326 °C. These changes in the glass transition temperature could be attributed to the fact that the inorganic fillers restrict the movements of polymer chains [49]. This behavior could be explained in terms of a stable interfacial interaction between the carbon nanotubes and the polymer chains. Results similar to those have also been observed in other polymer-inorganic mixed matrix systems, such as polymer-zeolites blends [45,50].

### 3.3. FTIR Analysis

**Figure 6:**
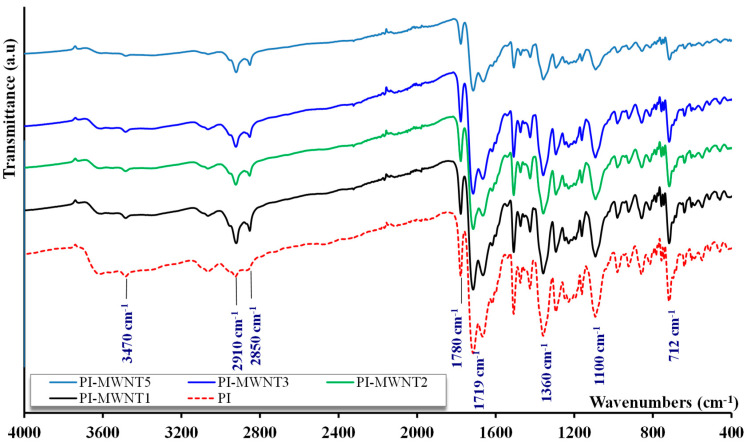
Absorption spectrum of samples PI, PI-MWNT1, PI-MWNT2, PI-MWNT3, and PI-MWNT5.

For the five studied membranes, characteristic absorption peaks of the functional groups of the membrane material were determined by FTIR. The characteristic absorption peaks for BTDA-TDI/MDI polymeric membrane matrix are marked in the spectra of Figure 6. 

The peak observed at 712 cm^−1^ is assigned to C–H (alkenes), whereas the next peaks at 1100 cm^−1^ and 1360 cm^−1^ exhibited are attributed to OC–N–CO (imide II). The peak at 1510 cm^−1^ is the characteristic absorption peak of O=C–N (amide II). The absorption peaks at 1719 cm^−1^ and 1780 cm^−1^ represent asymmetric OC–N–CO (imide I) [51,52]. A weak vibration at 3625 cm^−1^, corresponding to O–H (carboxylic acid groups), is observed where the carbonyl stretching of the acid group is overlapped by the absorption of the imidic carbonyl [53,54].

In addition, the symmetric and asymmetric stretching vibrations of CH_2_ groups give rise to the absorption peaks at 2910 and 2850 cm^−1^, respectively. This behavior in next nearest neighbor wavenumbers has also been reported for P84 polyimide film membranes [55]. A remarkable conclusion is that the addition of carbon nanotubes, at any of the studied concentrations, does not affect the chemical structure of the polyimide polymer matrix.

### 3.4. Mechanical Properties of MWCNTs/Polyimide Nanocomposite Films

Tensile experiments were carried out to determine the mechanical properties of the five studied membranes. The membranes were molded to dimensions of 2.5 cm by 15.0 cm and thickness of about 40 µm, left for 48 h at ambient conditions, and subsequently tensed at a crosshead rate of 50 mm/min until fracture.

**Figure 7:**
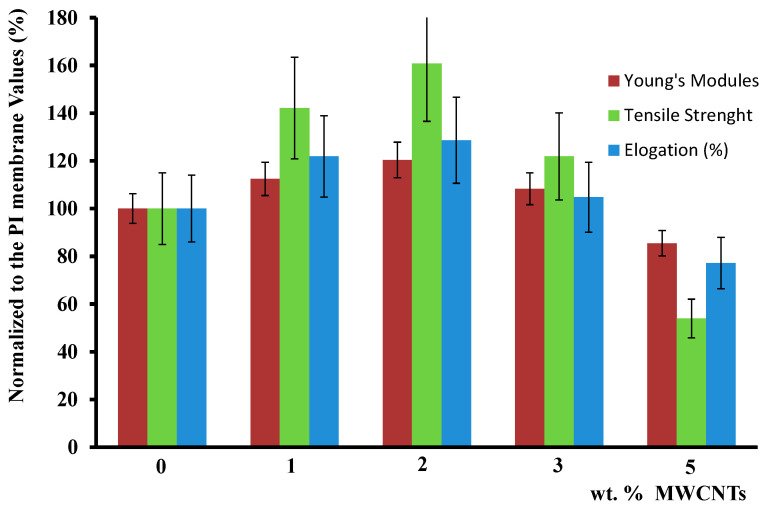
Relative representation of Young’s modulus, elongation, and ultimate tensile strength of tested PI samples as a function of MWCNTs concentration.

The findings from the mechanical tests indicated that 2% *w/w* was the optimum MWCNTs concentration and as it is shown in Figure 7 and Table 3, it had 28% higher elongation, 20% higher Young’s modulus, and 60% increase of the tensile strength with respect to neat PI membrane. This is a direct consequence of the fact that the best MWCNTs dispersion was obtained at this concentration, as previously shown by the electron microscopy images, and it is also confirmed from thermal and permeability measurements.

Figure 7 shows a maximum for the modulus of elasticity at 2% carbon nanotube loading probably because up to this concentration, there is “adequate” dispersion of the nanotubes in the polymer so to improve the mechanical properties. Well-dispersed nanotubes act as cross-linking points, resulting in an increase in the modulus of elasticity [56]. 

As the concentration of nanotubes in the polymer increased, their dispersion in the polymer matrix becomes more difficult resulting in aggregates that create weak points and increased mechanical stress, which in turn reduce the strength of the final polymeric membrane. Carbon nanotubes embedded in the polyimide matrix exhibit a behavior similar to the one observed with cross-linked polymer, thereby hindering the motion of the polymeric chains. Maximum elongation is observed at 2% carbon nanotubes loading. 

### 3.5. Gas Permeability Performance

For the dynamic oxygen permeability experiments under different relative humidity levels (0–85% r.h.) at 25 °C, membranes of 9 cm in diameter and ~40 µm in thickness were glued on paper sample holders, exposing an active membrane area of 42 cm^2^.

**Figure 8:**
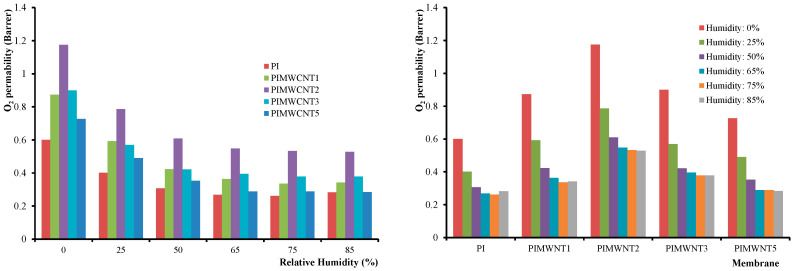
O_2_ permeability diagrams as a faction of relative humidity (**left**) and of different MWCNTs percentage into the mixed matrix polyimide membranes (**right**).

The measurements were performed in three specimens from each sample and their average was recorded. From the experimental results, as they are presented in Table 4 and Figure 8, it can be clearly observed that the PIMWNT2 membrane exhibits the maximum permeability. This could be explained by the formation of remarkable increased polymer free volume, formed between the polymer matrix and carbon nanotubes interface [34]. With a further increase of MWCNTs percentage, the nanocomposites seem to act as a barrier in the direction of diffused molecules. It can be concluded that the permeability follows the dissolution–diffusion model rather than the porous diffusion model. As expected, an increase in humidity levels led to a decrease of the O_2_ permeability since the water molecules act as barrier on the polymeric film.

For the implementation of permeability experiments using additional gases, membranes of 50 µm in thickness were cut to specimens of 3 cm in diameter and, subsequently, placed in the measuring apparatus. Prior to the gas permeability measurements, the samples were subject to degassing for 24 h at 100 °C at a high vacuum condition of about 10^−6^ mbar. Before degassing, the membranes were kept in a vacuum oven at 260 °C for about 5 h in order to stabilize the structure after the re-conformation of the polymeric chains and the packing of the fillers [57]. By this pre-treatment process, an improvement of the structural and mechanical stability of the mixed matrix membranes was achieved. The application of heating, below the glass transition, a process known as “annealing”, expedites the motion of the polymeric chains and provides the formation of denser and closer packing through the chains rearrangement [58]. By this way, minor defects in the precursor/pristine membrane can be rectified [59]. 

**Figure 9:**
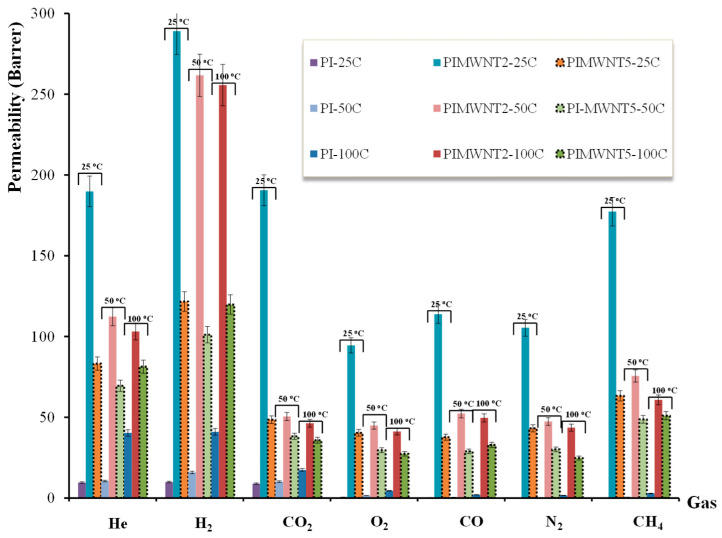
Permeability data of PI, PIMWNT2, and PIMWNT5 at 25, 50, and 100 °C for He, H_2_, CO_2_, O_2_, CO, N_2_, and CH_4_.

Figure 9 presents the permeation properties of He, H_2_, CO_2_, O_2_, CO, N_2_, and CH_4_, for the three selected polyimide based flat sheet membranes. By using the single gas permeability apparatus, which was described previously, the gas permeability was determined by using the equation:

(4) $$P=6\times 10^{8}\frac{V_{low}\times \left(\frac{\delta P_{low}}{\delta t}\right)\times l}{\Delta P\times U\times T_{exp}}\left(\mathrm{Barrer}\right)$$

where, *V_low_* is the collection volume (cm^3^), *δP_low_/δt* the pressure increase rate (mbar/min) in the collection volume, *U* the total active area of membrane sample (cm^2^), Δ*P* (mbar) the pressure head, *T_exp_* (K) the experimental temperature, and *l* (cm) the active layer thickness. The permeability of each studied gas through each membrane was measured for three runs and the presented values result from the average of these runs. For this kind of experiment, and taking oxygen permeability measurements into account, we decided to study the following membranes: PI, PIMWNT2, and PIMWNT5 at the temperatures of 25, 50, and 100 °C. This decision was supported by two reasons. First because the PIMWNT1 and PIMWNT3 provide similar oxygen permeability behavior and secondly for having exactly the same experimental conditions, during the gas permeability measurements, given the fact that the used apparatus consists of three membrane cells.

For a more comparative observation the gas permeability values of all studied gases, and for the selected PI, PIMWNT1, and PIMWNT5 membranes, are presented in Table 5. 

As clearly shown in Figure 9, and in agreement with the oxygen permeability measurements, at different humidity levels, the highest values are recorded for the PIMWNT2 membrane for all the studied gases. The gas permeability values from 0.1 Barrer for N_2_ at 25 °C and for the PI membrane up to 262 Barrer for H_2_ at 50 °C and for the PIMWNT2 membrane. The pure polyimide membrane provides, at 25 °C, permeability values of 9.6, 9.9, 8.9, 0.6, 0.3, 0.1, and 0.2 Barrer for He, H_2_, CO_2_, O_2_, CO, N_2_, and CH_4_, respectively.

**Figure 10:**
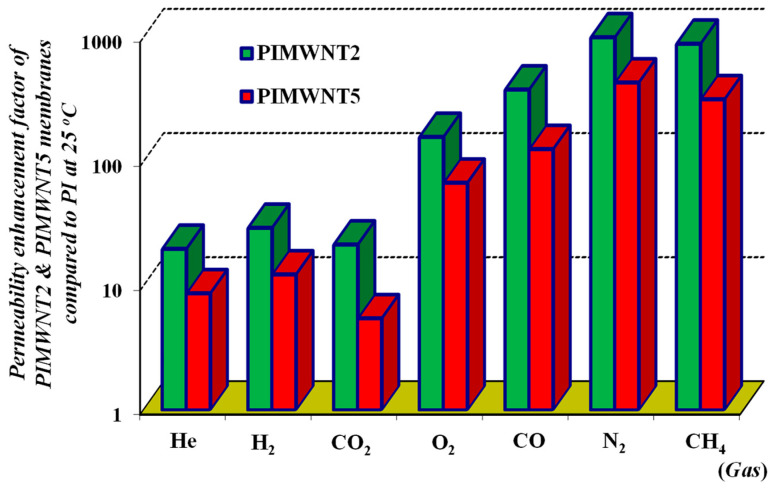
Permeability enhancement factor of PIMWNT2 (green area) and PIMWNT5 (red area) membranes compared to PI at 25 °C.

Similar permeability values for polyimide P84 flat sheet membranes are also reported in the literature [60,61]. In the case of the single polyimide membranes, the low values of gas permeability and the fact that the temperature has positive influence are the two main characteristics of the membranes’ behavior. On the other hand, by adding MWCNTs into the membrane matrix, there is an extremely large increase of the permeability, a behavior that is usually observed for lower permeable polymers, since the particles positively affect the gas separation properties of the mixed matrix membranes [62]. Specifically, the permeability of the PIMWNT2 and PIMWNT5 membranes, compared to PI membrane, increased from a factor of ~9 in the case of He up to a factor of 1054 in the case of N_2_ (see Figure 10). As it has been mentioned, the PIMWNT2 membrane is the one with the largest increase of the permeability values for all the studied gases. 

At the same time the influence of the temperature on the gas permeability behavior does not follow the mechanism of the temperature activation permeance. As it is shown in Figure 9 for both PIMWNT2 and PIMWNT5 membranes, by increasing the temperature the permeability decreases for all the studied gases. This means that the role of MWCNTs, in the membrane matrices, is to considerably reduce the positive dependence of the gas permeability on temperature and, finally, to provide an inversion of this trend. This behavior was observed with both membranes, these with filler concentrations of 2 and 5% wt and can be attributed to the fact that the carbon nanoparticles can hinder the polymer chain mobility. 

The addition of the filler nanomaterials lead to a decrease in polymer chain flexibility–mobility since the additives can act as a cross-linker. Since gas permeability through a polymeric matrix takes place mainly according to the solution–diffusion model in mixed matrix membranes the effect of the temperature change is less pronounced [63]. In particular, for the mixed matrix membranes PIMWNT2 and PIMWNT5 temperature increase led to a decrease in permeability for all studied gases, whereas in neat PI temperature increase led to an increase in permeability as expected. 

**Figure 11:**
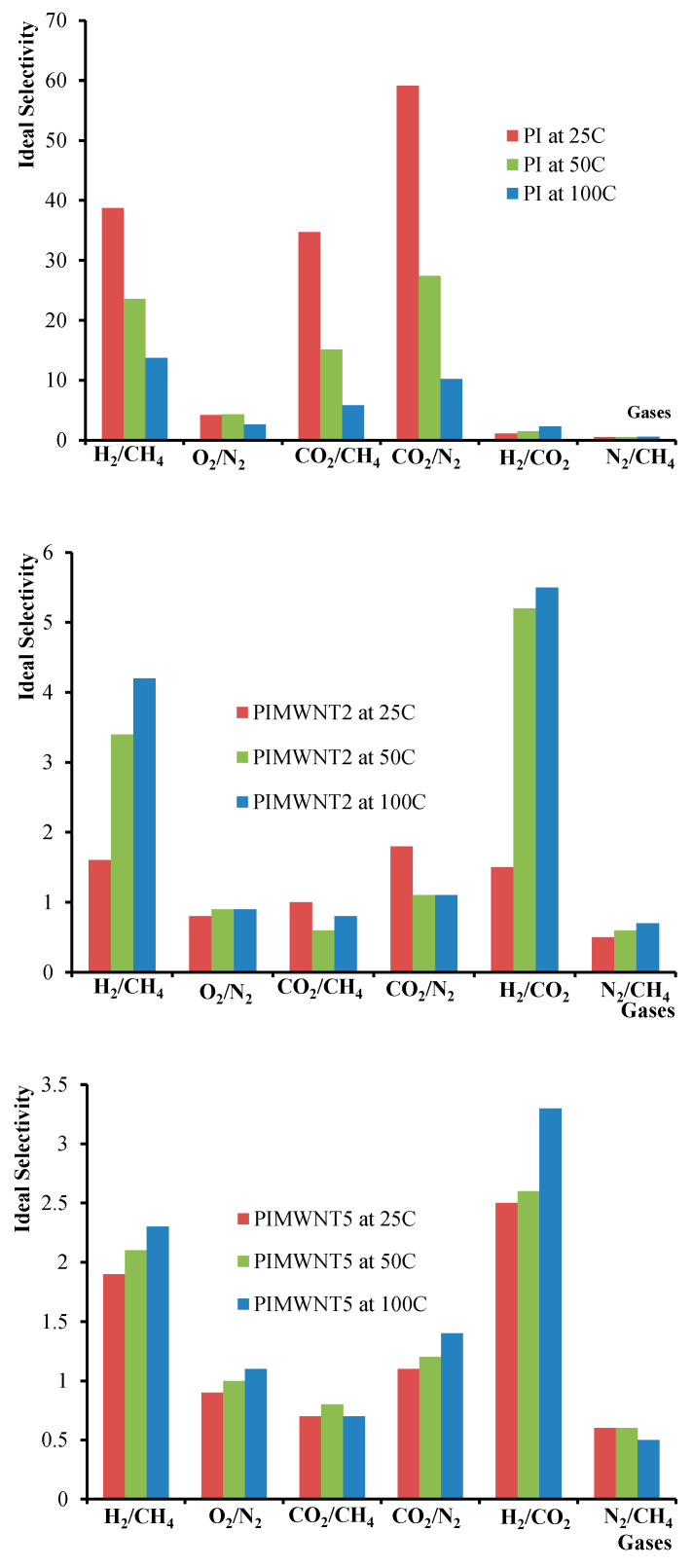

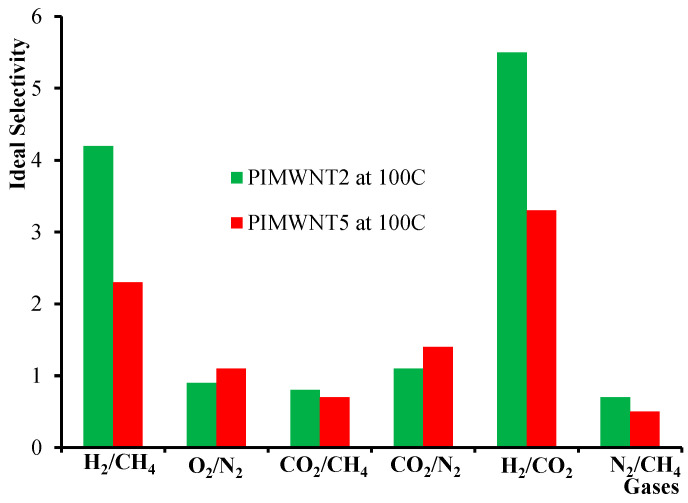
Ideal selectivities for PI, PIMWNT2, and PIMWNT5 membranes at 25, 50, and 100 °C corresponded to H_2_/CH_4_, O_2_/N_2_, CO_2_/CH_4_, CO_2_/N_2_, H_2_/CO_2_, and N_2_/CH_4_ pair of gases.

As depicted in Figure 11, the ideal selectivity values of the mixed matrix membranes are not significant. However, in the case of hydrogen separation, one could take advantage of the extremely high permeability values for the design of a membrane module as a promising unit for hydrogen separation tests. 

It is expected that the addition of MWCNT’s will create nano-micro level voids/cavities at the polymer interface region, but at the same time will introduce obstacles forcing the gas molecules to follow a tortuous path [64]. These two expected outcomes have opposite effects. Voids will increase the flux and reduce selectivity whereas the barrier acting particles will decrease permeability and depending on the final structure can also affect selectivity. 

Additionally, depending on the interaction between the polymeric chains and the carbon nanotube’s functional groups the membrane chemistry can also be altered. 

Based on the observation that for all the studied gases, the selectivity ratio of the nanocomposite membranes decreased in respect to the neat PI, it can be assumed that the permeation mechanism is not solely dependent on the solution–diffusion model but a portion also takes part in the void section of the membrane’s volume [65]. 

Remarkable differences in the achieved permeability properties for mixed matrix membranes are also reported in the literature. In specific, in our previous works, and by using the same system of MWCNTs as filler material and P84 polyimide as membrane matrix, we studied the effect of filler concentration on gas permeance properties in hollow fiber membranes configuration [23,32]. In this work an increase of gas permeances of two to five times was achieved by increasing the MWCNTs’ concentration up to 4% *w/w*. At the same time a decrease of ideal selectivity values of about four times was observed in the cases of He/CO_2_ and H_2_/CH_4_. A remarkable increase in H_2_, CH_4_, O_2_, N_2_, and CO_2_ permeability performance was also observed by Zang et al. [66]. In this work a wide range of SWCNT concentrations was used for the preparation of mixed matrix 6FDA-TP polyimide based flat sheet membranes. The authors used three different types of SWCNTs, as-purchased, purified, and acid-treated, and the differences of the gas permeability values, among the three derivative membranes, were negligible and always increased by three to four times compared to the net 6FDA-TP polyimide membrane. The calculated ideal gas selectivities for H_2_/CH_4_, H_2_/N_2_, and H_2_/CO_2_ were recorded smaller at about 1.5 to 2 times. Here it should be noted that in this work the A-SWNT/6FDA-TP membrane with 2% wt SWCNTs also exhibited both high permeabilities and selectivities compared to the net 6FDA-TP polyimide membrane.

Similar behavior was reported by Wang et al. [67], where covalently grafted polyetheramine (M2070)-carbon nanotubes or graphene oxide solvent-free hybrid nanofluids were embedded into Pebax-1657. By this effort, the authors achieved a CO_2_/N_2_ separation factor of 72 with a CO_2_ permeability of 332 Barrer at 1.0 bar and 25 °C under dry mixed feed gases state. In another study of Modi et al. [68], a remarkable increase of CO_2_ and O_2_ permeability, of about 10 times, was observed, whereas by adding carboxylated carbon nanotubes into polyethersulfone matrix insignificant changes were observed in CH_4_ and N_2_ as a consequence, a remarkable increase in CO_2_/CH_4_, O_2_/N_2_, and CO_2_/N_2_ selectivity was achieved. 

A different approach is reported by Habibiannejad et al. [69], where Pebax-1657 and Pebax-1657/modified MWCNTs mixed matrix membranes were investigated for O_2_, N_2_, and CO_2_ at different pressures and temperatures. In this work, the permeability of CO_2_ at 30 °C and 3 bar in the Pebax-Neat and Pebax-MWCNTs (-NH_2_ modified) membranes was the same, whereas the CO_2_/N_2_ selectivity of the Pebax-MWCNTs (-NH_2_ modified) was 125% more than that of the Pebax-Neat at the same condition. It was found that the gas separation performance of the prepared membranes remarkably depends on the properties of permeating gas molecules as well as the operating pressure and temperature. The main result of this study was that amine functionalized MWNT/Pebax and the 6% -COOH functionalized/Pebax showed outstanding performance on CO_2_/N_2_ separation at low working pressure and high working pressure, respectively. Many other works also refer to the effect of the filler material type and the concentration on the membrane on gas permeability/selectivity performance, while many different approaches are reported concerning the main mechanism contribution to the final mixed matrix membrane properties [70,71,72,73,74,75,76]. 

Here it could be noted that a remarkable conclusion of our current work is that although the contribution of the MWCNTs materials into the polyimide membrane matrix has negative effect on the gas ideal selectivity properties at the same time a vast increase of the gas permeability values was recorded, which fluctuates from around 100 to 1000 times the values of the neat polyimide membranes, for O_2_, CO, N_2_, and CH_4_ pure gases. 

Furthermore, the activation energy of gas permeation through the studied membranes was calculated and employed as a useful tool for understanding the “*grade of the spontaneity*” of the gas permeation mechanism. The activation energy for gas permeation (kJ/mol) is the sum of the activation energy for diffusion *E_d_* and the heat of sorption, also known as molar enthalpy of solvation, Δ*H_s_* (generally exothermic i.e., Δ*H_s_* takes negative values). Usually, increasing temperature enhances gas flow through a polymer matrix. As temperature rises, the gas diffusivity, in a membrane, increases, whereas its sorption coefficient decreases, causing variation in the permeability according to the following equation [77]:

(5) $$P_{i}=D_{i}\times S_{i}=\frac{Flux}{\Delta P_{i}/l}$$

where *D* is the diffusivity and *S* is the sorption coefficient of the gas penetrant “*i*” through the membrane, Δ*P_i_* is the partial pressure difference, and *l* is the membrane thickness. In the current work all gas permeability activation energies were determined from the slope of each Arrhenius plot by plotting the natural logarithm of flux, *J*, versus reciprocal temperature:

(6) $$J=J_{O}\times exp\left(\frac{E_{j}}{RT}\right)$$

where *E_j_* has been considered to be the activation energy for permeation [78]. An interesting fact is that the reported polymers which are defining the upper bound relationship in permeability versus selectivity Robeson’s plots, for light gas pairs, are amorphous and glassy, with stiff-chains [79]. BTDA-TDI/MDI (P84) is a typical glassy polymer. 

The gas permeability activation energies were calculated, for PI, at 8 kJ/mol for CO_2_, 35 kJ/mol for N_2_ and 33 kJ/mol for CH_4_, which are in agreement with literature reported values [80]. On the other hand, both for PIMWNT2 and PIMWNT5 membranes and for the CO_2_, N_2_, and CH_4_ gases, the permeability activation energy was found to be negative. Similar results were found in our previous work and only in the case of modified MWCNTs-based mixed matrix P84 hollow fiber membranes [32]. Negative values in activation energy of gas permeation have been reported for both various polymeric and inorganic membranes [81,82] and have been explained in terms of the negative influence of temperature on solubility and, thus, on permeation [83]. In addition, the existence of high concentration of the MWCNTs fillers provides a larger free volume within the polymer matrix resulting in negative activation energy of permeation because of the reduction of the flux resistance. 

## 4. Conclusions

In the present work, non-porous pure polyimide membranes and composite polyimide membranes with carbon nanotubes were studied. N-methyl-2-pyrrolidone was used in their preparation, whereas in the case of composite membranes the maximum concentration of carbon nanotubes was 5% wt. Thermal characterization methods (TGA and DSC) showed that the thermal properties of the polyimide membranes were improved by adding MWCNTs materials as membrane fillers. The optimum properties were obtained at a filler concentration of 2% wt. The study of the mechanical properties of the membranes revealed that the optimal performance was observed once again for the composite membrane PIMWNT2, and this was attributed to the effective dispersion of the nanotubes in the polymer at 2% wt filler concentration. By adding a larger percentage of nanotubes, the mechanical properties of the membranes deteriorated. The gas permeability results demonstrate that the PIMWNT2 membrane exhibits maximum permeability at all temperatures compared to the other composite membranes. In conclusion, the addition of carbon nanotubes to the polyimide membranes’ matrices improves its studied properties due to the attainment of uniform dispersion of the nanotubes in the polymer matrix which could play the role of a polymeric chains’ cross-linker. Adding more than 2% wt in carbon nanotubes to the polyimide polymer matrix results in the deterioration of the final properties of the polymeric membranes due to the formation of agglomerates. More than 5% wt of carbon nanotubes concentration does not favor the production of homogeneous polyimide membranes. The permeation mechanism is the result of the cooperativeness of the solution–diffusion mechanism of the gas through the polymeric matrix and the gas diffusion mechanism through the formatted polymeric free volume and/or filler–polymer interfaces. 

## Figures and Tables

**Table 1:** Material concentration, including multi-wall carbon nanotubes (MWCNTs), per prepared coded film.

Sample Code	Polyimide	MWCNTs
	wt.%	wt.%
PI	100	0
PIMWNT1	99	1
PIMWNT2	98	2
PIMWNT3	97	3
PIMWNT5	95	5

**Table 2:** Loss of membrane mass during pyrolysis at the maximum temperature and glass transition temperature of the polymer.

Membrane	PI	PIMWNT1	PIMWNT2	PIMWNT3	PIMWNT5
**Mass Loss (%) at 900 °C**	40.5	38.2	40.5	42	39.5

**Table 3:** Young’s modules, elongation, and tensile strength measurements of the five polyimide based studied membranes.

Sample	Young’s Modulus GPa	Elongation %	Tensile Strength MPa
PI	1.373 ± 0.09	3.98 ± 0.77	45.8 ± 7.50
PIMWNT1	1.544 ± 0.16	4.85 ± 0.17	65.1 ± 5.41
PIMWNT2	1.653 ± 0.18	5.12 ± 0.94	73.6 ± 6.11
PIMWNT3	1.487 ± 0.09	4.17 ± 0.56	55.8 ± 2.52
PIMWNT5	1.173 ± 0.08	3.07 ± 0.42	24.7 ± 3.61

**Table 4:** O_2_ permeability values at different humidity levels.

Relative Humidity (%)	Permeability (Barrer)				
	Membrane				
	PI	PIMWNT1	PIMWNT2	PIMWNT3	PIMWNT5
0	0.60 ± 0.014	0.87 ± 0.023	1.18 ± 0.027	0.90 ± 0.022	0.72 ± 0.030
25	0.40 ± 0.008	0.59 ± 0.017	0.78 ± 0.023	0.57 ± 0.015	0.49 ± 0.009
50	0.31 ± 0.009	0.42 ± 0.008	0.61 ± 0.024	0.42 ± 0.011	0.35 ± 0.007
65	0.27 ± 0.007	0.36 ± 0.009	0.55 ± 0.017	0.40 ± 0.098	0.29 ± 0.007
75	0.26 ± 0.010	0.34 ± 0.008	0.53 ± 0.015	0.38 ± 0.011	0.29 ± 0.006
85	0.28 ± 0.007	0.34 ± 0.009	0.53 ± 0.014	0.38 ± 0.010	0.28 ± 0.007

**Table 5:** Gas permeability values at different at different temperatures.

GAS	Permeability (Barrer)								
	25 °C			50 °C			100 °C		
	PI	PIMWNT2	PIMWNT5	PI	PIMWNT2	PIMWNT5	PI	PIMWNT2	PIMWNT5
He	9.6	189.8	83.2	10.6	112.3	69.5	40.3	103.1	81.3
H_2_	9.9	289	121.7	15.9	261.7	101.1	41	255.6	119.8
CO_2_	8.9	190.5	48.5	10.2	50.5	38.2	17.4	46.3	35.8
O_2_	0.6	94.5	40.4	1.6	44.9	29.6	4.5	41.2	27.4
CO	0.3	113.8	37.7	0.5	52.3	28.9	2.1	49.6	32.9
N_2_	0.1	105.4	43.2	0.3	47.4	30.1	1.7	43.6	24.8
CH_4_	0.2	177.3	63.3	0.6	75.6	48.7	2.9	60.8	51
